# Solvatochromic Study of Two Carbanion Monosubstituted 4-Tolyl-1,2,4-triazol-1-ium Phenacylids in Binary Hydroxyl Solvent Mixtures †

**DOI:** 10.3390/molecules26133910

**Published:** 2021-06-26

**Authors:** Dana Ortansa Dorohoi, Dan-Gheorghe Dimitriu, Mihaela Maria Dulcescu-Oprea, Ana Cezarina Morosanu, Nicoleta Puica-Melniciuc, Elena Ardelean, Antonina Gritco-Todirascu, Corina Cheptea

**Affiliations:** 1 Faculty of Physics, Alexandru Ioan Cuza University, 700506 Iasi, Romania; ddorohoi@uaic.ro (D.O.D.); opreamihaelamaria@yahoo.com (M.M.D.-O.); cezarina_morosanu@yahoo.com (A.C.M.); antoninagritco@yahoo.com (A.G.-T.); 2 Regional Institute of Oncology, 700483 Iasi, Romania; 3 Faculty of Orthodox Theology, Alexandru Ioan Cuza University, 700066 Iasi, Romania; nicoleta.melniciuc@uaic.ro (N.P.-M.); elena.ardelean@uaic.ro (E.A.); 4 Department of Biomedical Sciences, Faculty of Biomedical Engineering, “Grigore T. Popa” University of Medicine and Pharmacy, 700454 Iasi, Romania; corina.cheptea@umfiasi.ro

**Keywords:** carbanion monosubstituted 4-tolyl-1,2,4-triazol-1-ium phenacylids, solvatochromism, hydroxylic solutions, interaction energy in molecular pairs ylid-water and ylid-alcohol

## Abstract

Two 4-tolyl-1,2,4-triazol-1-ium methylids, namely 4-tolyl-1,2,4-triazol-1-ium-phenacylid and 4-tolyl-1,2,4-triazol-1-ium-4′-nitro-phenacylid, are studied from solvatochromic point of view in binary solvent mixtures of water with ethanol and water with methanol. The contributions (expressed in percent) of the universal and specific interactions are separated from the spectral shifts recorded in the visible range for each composition of the binary solvent mixture. The essential role of the orientation and induction interactions in the studied solutions was demonstrated. Based on the statistic cell model of the binary solvent mixture solutions, the difference between the formation energies of ylid-water and ylid-alcohol complexes is estimated. The composition of the ylid’s first solvation shell was also established using the model of the binary solvent mixture solutions. The results obtained from the statistical cell model were compared with those obtained by using the Suppan’s model, resulting a good agreement.

## 1. Introduction

Ylids are chemical compounds having a molecule containing a negatively charged carbon atom (called carbanion) directly bonded to a positively charged atom of nitrogen, phosphorus, sulfur, or another element [1,2]. The result can be seen as a structure in which two adjacent atoms are connected by both a covalent and an ionic bond. Thus, the ylids are a subclass of zwitterionic compounds.

**Figure 1:**
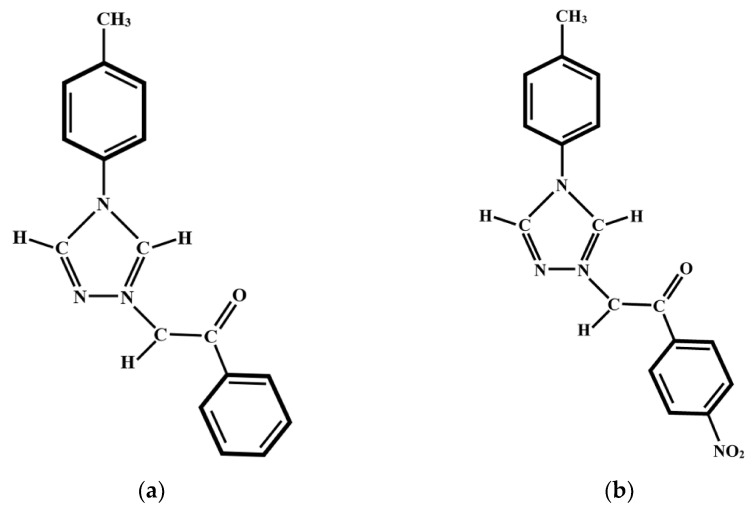
Chemical structure of 4-tolyl-1,2,4-triazol-1-ium phenacylid (TTPY) (**a**) and 4-tolyl-1,2,4-triazol-1-ium-4′-nitro-phenacylid (TTNPY) (**b**) molecules.

Two carbanion monosubstituted triazolium ylids [1,2], namely 4-tolyl-1,2,4-triazol-1-ium phenacylid (TTPY) and 4-tolyl-1,2,4-triazol-1-ium-4′-nitro-phenacylid (TTNPY) are considered in this study as spectrally active molecules. These are dipolar and polarizable molecules with zwitterionic character, having opposite charges separated on one nitrogen of the triazolium cycle and on the negative, monosubstituted carbanion, respectively. Figure 1 shows the chemical structure of the two compounds.

The pharmacological activity and clinical implications of triazolium agents were emphasized in some reviews [3,4]. The chemical and biological applications of triazolium ylids or of their derivatives were also analyzed in numerous publications [5,6,7,8,9].

The knowledge about the interactions of triazolium ylids with different solvents are important because all reactions of these compounds take place in situ. Previous studies revealed the nature of the intermolecular interactions of some cycloimmonium ylids with the liquids in which they are dissolved, based on the solvatochromic analysis of their visible band and very sensitive to the solvent action [10,11,12].

Taking into account the basic character of cycloimmonium ylids and the solvatochromic analysis, this results in their affinity to form hydrogen bonds with hydroxylic molecules by acceptance of protons. The study of binary solvent mixture solutions of some cycloimmonium ylids can offer the opportunity to characterize the strength of the hydrogen bond between the ylid and hydroxylic solvent molecules, as it was made in previous studies for another ylids [11,12].

1,2,4-Triazolium methylids belong to cycloimmonium class and are basic compounds. They show a visible electronic absorption band [13,14,15,16,17], relatively sensible to the solvent action. As it is known [13,14], triazolium ylids are a few soluble in most of the solvents. Therefore, one solvatochromic study of these molecules in a great number of liquids is practically impossible. They were studied in a few numbers of solvents and in some binary solvent mixtures made by one hydroxylic and one non-hydroxylic liquids [14,15,16,17]. 1,2,4-Triazolium methylids are relatively soluble in water and alcohols, and their spectral study in binary solvent mixtures realized from water and one primary alcohol becomes interesting.

The spectral UV-Vis data are very important for understanding the behavior of the ylids in biomedical processes, i.e., for their applications. For example, in quantum mechanics, the biological properties of a substance are correlated with the energy gap $\Delta E=hc\bar{\nu }$ where $\bar{\nu }$ is the wavenumber corresponding to the maximum of the electronic absorption band of the molecule. For small values of Δ*E*, the substances are more reactive.

In this spectral study, we intend to apply the statistical cell model of binary solvent mixture solutions to the solutions of 1,2,4-Triazolium methylids in mixtures water (1) + ethanol (2), both liquids being of biological interest. For comparison, we added the similar solvatochromic study for the same ylids in binary solvent mixture water (1) + methanol (2).

## 2. Calculations and Models

The Kamlet–Abboud–Taft (KAT) parameters [18,19] are known for water and the primary alcohols and for their mixtures [20], and can be used in solvatochromic studies. Such study is difficult to achieve because the binary solvent mixtures were made step by step with a 0.05 molar fraction and the solubility of the studied ylid is reduced even in the mixtures of water and different alcohols, but it can offer information about the structure of the binary solvent mixture solutions of the studied ylids.

The solvatochromic analysis in binary solvent mixture solutions offers information about the nature of the intermolecular interactions between the solute and solvent molecules and about the composition of the first shell of the solute molecules.

Some correlations [21,22,23] between the wavenumber in the maximum of the electronic absorption band and the solvent parameters, such as the hydrogen bond donor (HBD, noted by *α*), hydrogen bond acceptor (HBA, noted by *β*), and polarity/dipolarity number ($\pi^{*}$), known as KAT parameters, were established:

(1) $$\bar{\nu }\left({\mathrm{cm}}^{-1}\right)={\bar{\nu }}_{0}\left({\mathrm{cm}}^{-1}\right)+C_{1}\pi^{*}+C_{2}\alpha +C_{3}\beta$$

The correlation coefficients ${\bar{\nu }}_{0}$ and *C*_1_–*C*_3_ in Equation (1) can be estimated by statistical analysis of the experimental data. They give by their sign and value the sense and the contribution of intermolecular interactions to the electronic absorption band shift in solutions relative to its position in the gaseous phase of the ylid. The contribution of each type of intermolecular interactions to the spectral shift in each binary solvent mixture can also be established based on the values obtained for the regression coefficients *C*_1_–*C*_3_ and the values of the solvent parameters.

The obtained results in this research can also be judged on the basis of the statistical cell model of the binary solvent mixture solutions [24,25,26,27,28,29]. In the binary solvent mixture solutions studied by us, the ylid molecules can participate in hydrogen bonds with both solvents, but the formation energies of the complexes differ for the molecules of water, ethanol, or methanol.

In the statistical cell model, the wavenumber $\bar{\nu }$ (cm^−1^) in the maximum of the absorption band can be computed using the relative statistical average weights *p*_1_ and *p*_2_, being *p*_1_ + *p*_2_ = 1, of the binary solvent mixture (made by water (1) considered the most active solvent and ethanol/methanol (2) considered the less active solvent) [24,25].

(2) $${\bar{\nu }}_{t}=p_{1}{\bar{\nu }}_{1}+\left(1-p_{1}\right){\bar{\nu }}_{2}$$

In relation (2), the indices t, 1, and 2 refer to binary solvent mixtures and binary solutions made in solvent (1) and (2), respectively. Relation (2) shows that the average statistic weights *p*_1_ and *p*_2_ of the two liquids in the first solvation shell of ylid can be computed using the wavenumbers in the maximum of the visible electronic band in binary solvent mixture solution at each molar fractions *x*_1_ and *x*_2_, being *x*_1_ + *x*_2_ = 1, of the binary solvent mixture. Knowing the bulk molar fractions *x*_1_ and *x*_2_ of the two solvents in the mixture, the statistic cell model permits to compute the average statistic weights, *p*_1_ and *p*_2_, and to establish the following relation between these parameters [24,25,26,27,28]:

(3) $$\ln\frac{p_{1}}{1-p_{1}}=\ln\frac{x_{1}}{1-x_{1}}+n$$

In relation (3) the cut at origin *n* depends on the difference *w*_2_ − *w*_1_ between the interactions in molecular pairs of the type: ylid—water and ylid—alcohol. This dependence is given in relation (4), where *k* is the Boltzmann constant and *T* is the absolute temperature:

(4) $$n=\frac{w_{2}-w_{1}}{kT}$$

Based on the cell statistic model of the binary solvent mixture solutions, the difference *w*_2_ − *w*_1_ can be estimated using the spectral data [26,27,28]. This difference is hard to determine with other methods in common laboratories.

The statistical cell model of the binary solvent mixture solutions allows the estimation of the composition of the ylid first shell in the binary solvent mixture solution by the parameter $\delta_{1}=p_{1}-x_{1}$, called the excess function of solvent (1) in the first solvation shell, or by $K_{12}=\frac{p_{1}}{p_{2}}\frac{x_{2}}{x_{1}}$, called the preferential solvation constant [29]. When *δ*_1_ > 0, or *K*_12_ > 1, the active solvent noted by (1) is dominant in the first shell of the solute, while the solvent (2) is predominant in the first solvation shell of the solute when the inequalities are inverse.

Similar to the statistical cell model, the model of Suppan [30,31,32] describes the preferential solvation of solutes in binary solvent mixtures, being frequently approached in the last years [33,34,35,36]. The basic idea is that, in a binary solvent mixture, the dipolar solute will be preferentially solvated by the most dipolar solvent. Consequently, the mole fraction in the cybotactic region will be higher for this solvent, i.e., the mole fractions of the two solvents in the cybotactic region differ from those ones in the bulk solution. The ratio of the mole fractions in the cybotactic region (*y*_2_/*y*_1_) is proportional to the ratio of the mole fractions in the bulk solution (*x*_2_/*x*_1_) according to Equation (5):

(5) $$\frac{y_{2}}{y_{1}}=\frac{x_{2}}{x_{1}}e^{-Z}$$

In the above equation, *Z* is the so-called index of preferential solvation [37], and it can be estimated from spectrochemical data through the next equation:

(6) $$\frac{1}{\Delta E_{CT}}=-\frac{2a^{3}}{\mu^{2}\Delta \varphi {\left(\varepsilon \right)}_{1-2}}\left(1+\frac{x_{2}}{x_{1}}e^{-Z}\right)$$

where

(7) $$\varphi \left(\varepsilon \right)=\frac{2\left(\varepsilon -1\right)}{2\varepsilon +1}$$

and *ε* is the dielectric constant, $\Delta E_{CT}=hc\left({\bar{\nu }}_{t}-{\bar{\nu }}_{2}\right)$ is the inverse peak shift (measured against the peak position corresponding to the solvent 2), and *μ* and *a* are the dipole moment and the molecular radius of the solute molecule, respectively.

If the specific solute–solvent interactions have a negligible contribution to the solvatochromism, the graphic representation of 1/Δ*E_CT_* versus *x*_2_/*x*_1_ will be a straight line. On the contrary, if the specific interactions are important, the graphical representation will show large deviations from linearity [30,31,32,33,34,35,36].

Such studies offer a better understanding of the mechanisms of intermolecular interactions in binary solvent mixtures.

## 3. Results and Discussion

**Figure 2:**
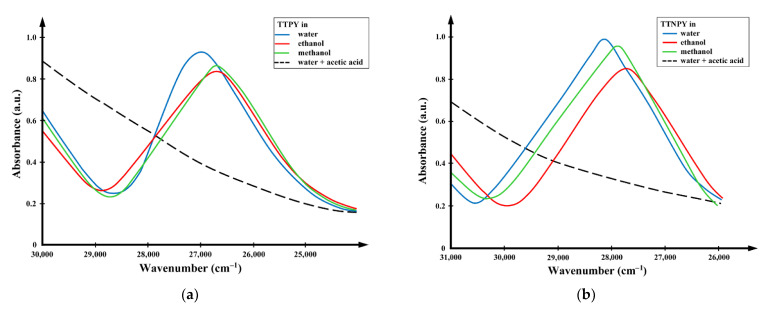
Spectra of 4-tolyl-1,2,4-triazol-1-ium phenacylid (TTPY) (**a**) and 4-tolyl-1,2,4-triazol-1-ium-4′-nitro-phenacylid (TTNPY) (**b**) in water, ethanol, methanol, and water + acetic acid, respectively.

Figure 2 shows the spectra of TTPY and TTNPY in water, ethanol, methanol, and water + acetic acid, respectively. The quenching of the visible electronic absorption band in the presence of the acetic acid proves that this band appears by *n* → π* transitions of the non-participant electron pair of the carbanion towards the heterocycle or towards the carbanion substituent.

The binary solvent mixtures parameters polarity/polarizability, π* (characterizing the universal interactions between the solute molecule and all solvent molecules), HBD number *α*, and HBA number *β* (characterizing the specific interactions of the hydrogen bond type by donating and receiving proton by a solvent molecule, respectively) determined by Buhvestov et al. [20], as well as the measured wavenumbers in the maximum of the visible bands of TTPY and TTNPY, are listed in Table 1 and Table 2 for the binary hydroxylic solvent mixtures: water (1) + ethanol (2) and water (1) + methanol (2), in various water molar concentrations, *x*_1_.

An increase in the wavenumber of the visible band of the studied ylids (TTPY and TTNPY) is emphasized in Table 1 and Table 2 when the water content increases in the binary solvent mixture water (1) + alcohol (2), both for the solvent achieved with ethanol and methanol. The same tendency has been observed in the binary solvent mixture solutions of the carbanion monosubstituted *p*-phenyl-triazol-1-ium ylids [23]. This tendency could be explained by the increase of the electric permittivity of the binary solvent mixture when its content in water increases, determining the increase of the universal interactions of the orientation–induction type in solutions.

The experimental data from Table 1 and Table 2 were subjected to statistical analysis based on relation (1), and the coefficients multiplying the solvent parameters were estimated (see Table 3) by using the application Multiple Linear Regression from OriginPro 9 software. The coefficients *C*_j_ and j = 1,2,3, multiplying the solvent parameters π*, *α*, and *β*, are listed in Table 3. They give by their value and sign the magnitude and the sense of the spectral shifts induced by the binary solvent mixture in the visible electronic spectra of the studied ylids. As relation (1) suggests, the sign plus of the correlation coefficients reflects a spectral shift to high wavenumbers (blue shift) in the electronic spectra. All coefficients are positive, showing a hypsochromic effect both of universal interactions (described by the π*, the polarity/polarizability parameter of the solvent) and of the specific interactions (described by the solvent parameters *α* and *β*) [21,22,23]. This tendency has been observed in other binary hydroxylic solvent mixture solutions of some cycloimmonium ylids [22,38].

Based on relation (1), the values of the regression coefficients from Table 3, and the binary solvent mixture parameters from Table 1 and Table 2, the contribution (expressed in percent) of each type of intermolecular interaction to the spectral shift can be estimated. The results of computations are listed in Table 4 and Table 5.

**Figure 3:**
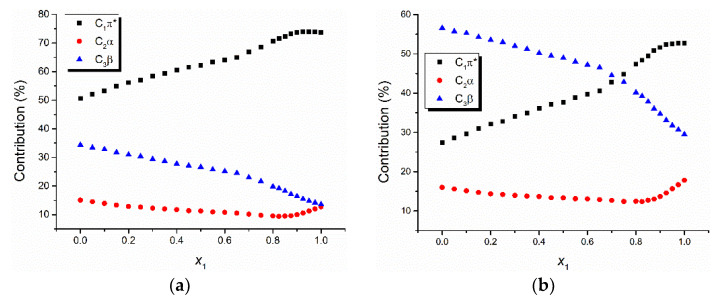
The contribution (percentage) to the spectral shift of universal and specific interactions for 4-tolyl-1,2,4-triazol-1-ium phenacylid (TTPY) (**a**) and 4-tolyl-1,2,4-triazol-1-ium-4′-nitro-phenacylid (TTNPY) (**b**) in binary solvent mixture water + ethanol.

**Figure 4:**
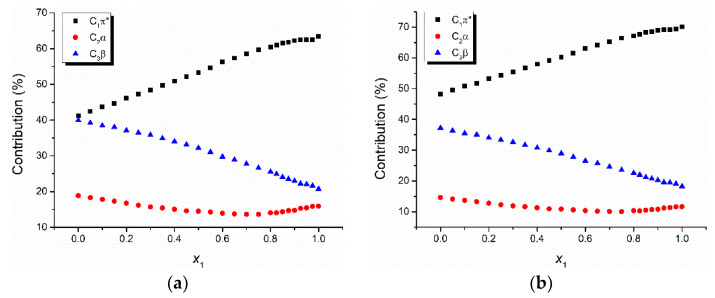
The contribution (percentage) to the spectral shift of universal and specific interactions for 4-tolyl-1,2,4-triazol-1-ium phenacylid (TTPY) (**a**) and 4-tolyl-1,2,4-triazol-1-ium-4′-nitro-phenacylid (TTNPY) (**b**) in binary solvent mixture water + methanol.

If the data from Table 4 and Table 5 are plotted in bidimensional graphs versus the water molar concentration, one obtains Figure 3 and Figure 4 for the two binary solvent mixtures: water + ethanol and water + methanol.

The universal interactions determine the highest shift of the visible electronic absorption band of the studied triazolium ylids in most of the studied solutions, except for the solution TTNPY + water + ethanol, where the specific interactions described by the term *C*_3_*β* are dominant for small values of the water content (*x*_1_ = 0–0.7). The contribution of the universal interactions (described by the term *C*_1_π* in relation (1)) increases with the water content in the binary solvent mixture (Figure 3 and Figure 4). The specific interactions described by the term *C*_2_*α* were the weakest. They contributed to the spectral shift of the visible electronic absorption band by a percent smaller than 20%. This observation could be explained by the fact that in all studied solutions, the ylid molecules (TTPY and TTNPY) formed complexes by hydrogen bonding. Though the probability of the hydrogen bonds in which the solvent receives protons from ylid (described by the term *C*_3_*β*) is small (except for the solution TTNPY + water + ethanol for *x*_1_ = 0–0.7), the contribution of these interactions to the total spectral shift is more important than the contribution of the specific interactions in which the ylid molecules (TTPY and TTNPY) receive protons. The contribution of the term *C*_3_*β* to the spectral shift of TTPY and TTNPY visible absorption bands decreased with the increase of the water content in the binary solvent mixture.

The decrease of the contribution of specific interactions to the total spectral shift at a high content of water in binary solvent mixture solutions of triazolium methylids has been explained by the presence of water complexes formed in these solutions [20].

**Figure 5:**
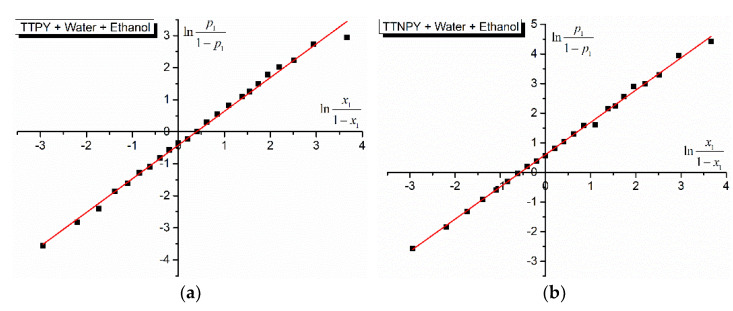
$\ln\frac{p_{1}}{1-p_{1}}$ vs. $\ln\frac{x_{1}}{1-x_{1}}$ for 4-tolyl-1,2,4-triazol-1-ium phenacylid (TTPY) (**a**) and 4-tolyl-1,2,4-triazol-1-ium-4′-nitro-phenacylid (TTNPY) (**b**) in binary solvent mixture water (1) + ethanol (2).

**Figure 6:**
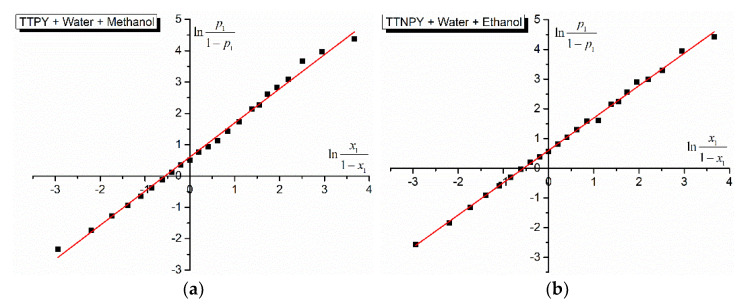
$\ln\frac{p_{1}}{1-p_{1}}$ vs. $\ln\frac{x_{1}}{1-x_{1}}$ for 4-tolyl-1,2,4-triazol-1-ium phenacylid (TTPY) (**a**) and 4-tolyl-1,2,4-triazol-1-ium-4′-nitro-phenacylid (TTNPY) (**b**) in binary solvent mixture water (1) + methanol (2).

The cell model of the binary solvent mixture solutions applied to the studied tolyl-triazol-1-ium phenacylids offers information about the difference between the potential energies in molecular pairs of the type ylid-water and ylid-alcohol. The dependences $\ln\frac{p_{1}}{1-p_{1}}$ vs. $\ln\frac{x_{1}}{1-x_{1}}$ are illustrated in Figure 5 and Figure 6.

The characteristics of the lines in Figure 5 and Figure 6, obtained by using the application Linear Fit from OriginPro 9 software, are given in Table 6. The slopes of the lines (3) are near the unity, as the cell model of the binary solvent mixture solutions predicts. The cut at origin gives the strength of the hydrogen bonds in the studied solutions by the difference *w*_2_ − *w*_1_ between the formation energies of the complexes ylid (TTPY/TTNPY)-water and ylid (TTPY/TTNPY)-alcohol. In the last column of Table 6, this difference is expressed in kJ/mol. The values from Table 6 show weak differences between the formation energies of hydrogen bonds between TTPY/TTNPY and the hydroxylic components of the solution [11,17,25]. Similar results (of the same order of magnitude) were obtained for the binary hydroxylic solvent mixtures water + ethanol and water + methanol and different probes in previous studies [15,17,22,38].

As can be seen from Figure 5 and Figure 6 and Table 6, a good linear dependence exists between $\ln\frac{p_{1}}{1-p_{1}}$ and $\ln\frac{x_{1}}{1-x_{1}}$, demonstrating the applicability of the statistic model of binary solvent mixture solutions achieved with the studied triazolium ylids (TTPY and TTNPY) and giving us the opportunity to evaluate the differences between the potential energies in molecular pairs realized by hydrogen bonds in the studied solutions (see Table 6). In the case of TTPY dissolved in water and ethanol, the difference *w*_2_ − *w*_1_ was negative, showing that |*w*_2_| > |*w*_1_| (see Figure 6a). The hydrogen bond of TTPY with ethanol was stronger than that between TTPY and water molecules. For the other solutions, the differences *w*_2_ − *w*_1_ were positive, showing that the hydrogen bond TTPY–water was stronger than the hydrogen bond TTPY–methanol, and the hydrogen bond TTNPY–water was stronger than TTNPY–ethanol and TTNPY–methanol.

**Figure 7:**
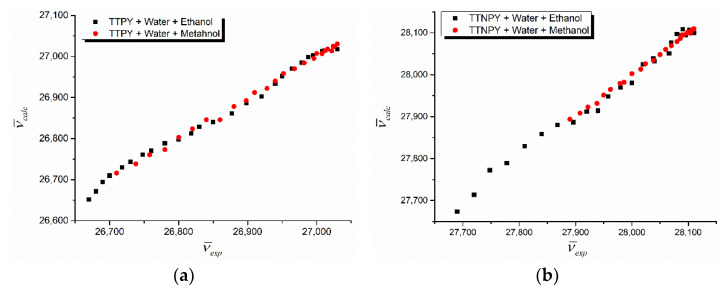
Computed wavenumber vs. experimental wavenumber for binary solvent mixture solutions of 4-tolyl-1,2,4-triazol-1-ium phenacylid (TTPY) (**a**) and 4-tolyl-1,2,4-triazol-1-ium-4′-nitro-phenacylid (TTNPY) (**b**).

The computed wavenumbers based on relation (1) and using the regression coefficients from Table 3 are plotted versus the corresponding experimental values in Figure 7. As is shown in Figure 7, the obtained curves reflect some changes in TTPY solutions by passing from the complexes formed in alcohol to the complexes formed in water molecules, when the water content increases.

In the studied solutions, the hydrogen bonds were very weak [12,17], and the thermal motion can change the nature of the complexes. At high water content, the complexes of the TTPY-water and TTNPY-water were dominant, and the water cages determined their relative stability [20].

**Figure 8:**
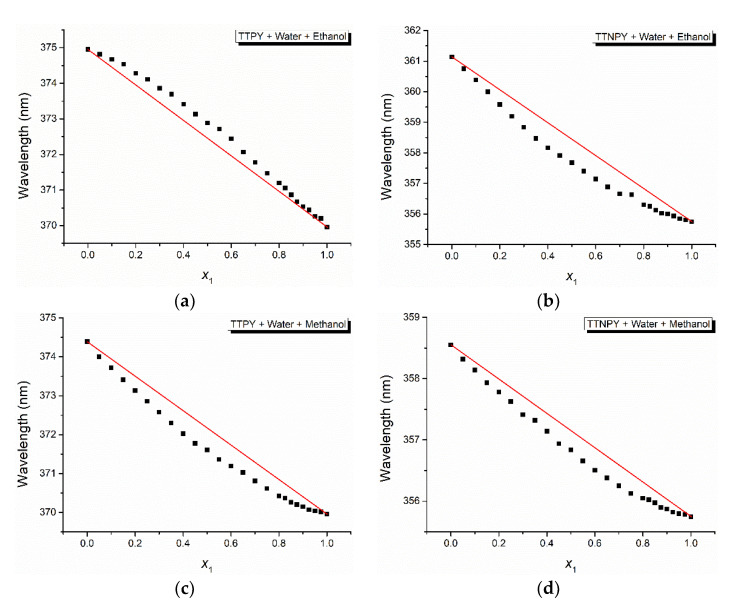
Wavelength in the maximum of the electronic absorption bands versus the water content for the investigated binary solvent mixture solutions of 4-tolyl-1,2,4-triazol-1-ium phenacylid (TTPY) (**a**,**c**) and 4-tolyl-1,2,4-triazol-1-ium-4′-nitro-phenacylid (TTNPY) (**b**,**d**).

**Figure 9:**
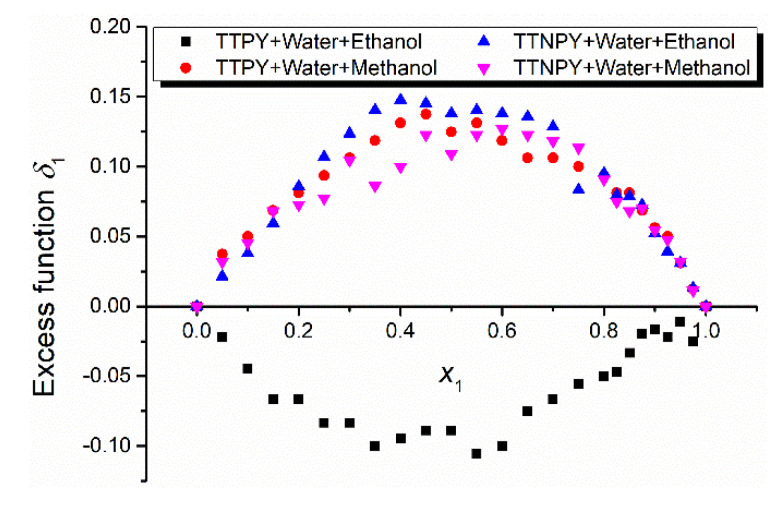
Excess function of the water molecules versus the water content in solution.

The cell model of binary solvent mixture solution allows the estimation of the composition of TTPY and TTNPY, respectively (first shell). Figure 8 shows the dependence of the wavelength in the maximum of the electronic absorption band on the water content *x*_1_. Figure 9 shows the excess function *δ*_1_ = *p*_1_ − *x*_1_ of the water at the increase of its content. Except for the solution TTPY + water + ethanol, where the ethanol molecules are predominant in the first solvation shell of TTPY molecule, the water molecules predominate.

**Figure 10:**
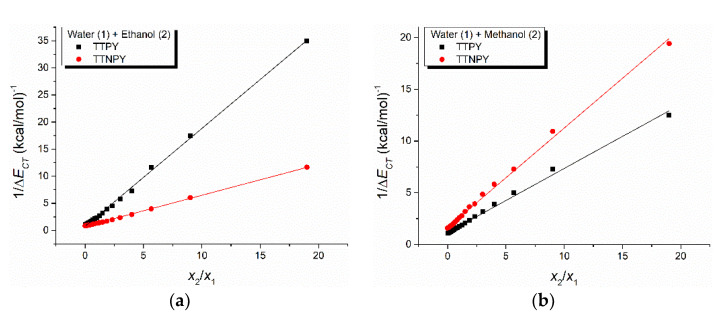
Inverse peak shift (measured against the peak corresponding to alcohols) of the 4-tolyl-1,2,4-triazol-1-ium phenacylid (TTPY) and 4-tolyl-1,2,4-triazol-1-ium-4′-nitro-phenacylid (TTNPY) absorption, respectively, versus the solvent bulk composition *x*_2_/*x*_1_, for the binary solvent mixtures water (1) + ethanol (2) (**a**) and water (1) + methanol (2) (**b**).

Figure 10 shows the dependence of 1/Δ*E_CT_* versus *x*_2_/*x*_1_ for TTPY and TTNPY in binary solvent mixtures water (1) + ethanol (2) (Figure 10a) and water (1) + methanol (2) (Figure 10b), respectively. The details of the linear fits (obtained by using the application Linear Fit from OriginPro 9 software) are given in Table 7. The very good linear dependence of 1/Δ*E_CT_* versus *x*_2_/*x*_1_ (R > 0.99, see Table 7) confirms that the hydrogen bonds were very weak, having a negligible influence on the spectral shift of the visible electronic absorption band. The values obtained for the index of preferential solvation (*Z*) can be compared with the values of the intercept (*n*) in the Equation (3), given in Table 6. Thus, all of the values were of the same order of magnitude and, moreover, a very good quantitative agreement was observed for the case of the binary solvent mixture water + methanol.

**Figure 11:**
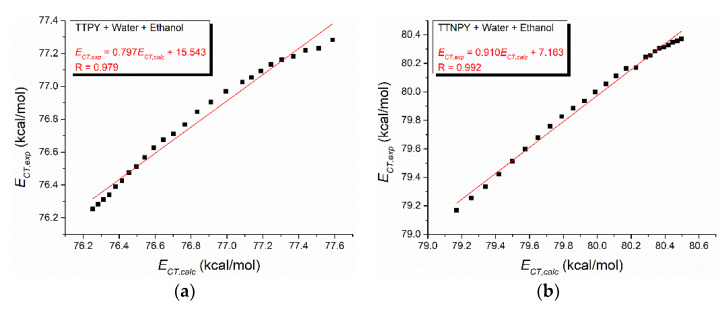

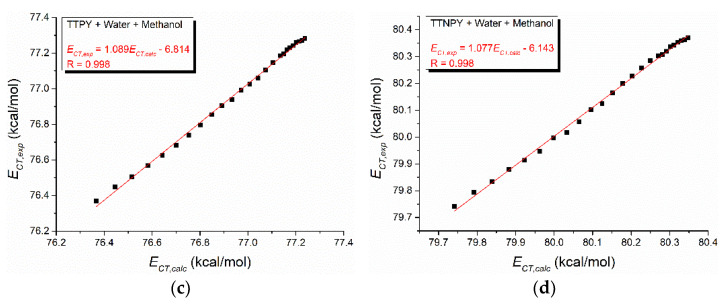
Experimental versus calculated charge transfer energies for 4-tolyl-1,2,4-triazol-1-ium phenacylid (TTPY) (**a**,**c**) and 4-tolyl-1,2,4-triazol-1-ium-4′-nitro-phenacylid (TTNPY) (**b**,**d**) in the binary solvent mixtures water + ethanol (**a**,**b**) and water + methanol (**c**,**d**).

Suppan’s model predicts the charge transfer energies with a high accuracy for the binary solvent mixture water + methanol, as can be observed in Figure 11, where the experimental versus calculated charge transfer energies are shown (R > 0.99 for both linear fits). In the case of the binary solvent mixture water + ethanol, Suppan’s model predicts with accuracy the charge transfer energies for TTNPY, but fails in the case of TTPY (slope = 0.79).

**Figure 12:**
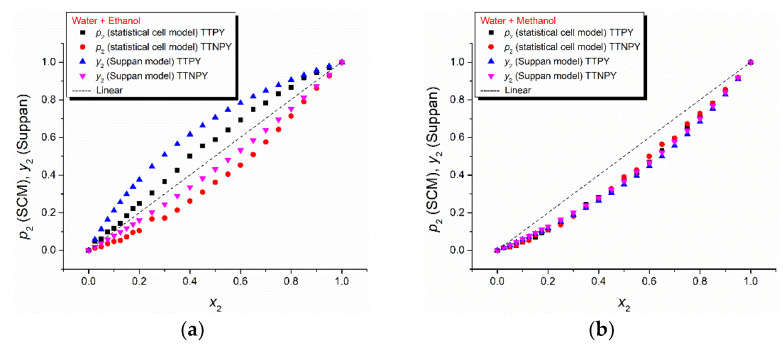
Local mole fractions calculated through statistical cell model (SCM) (*p*_2_) and Suppan’s model (*y*_2_) as a function of bulk mole fraction of ethanol (**a**) and methanol (**b**), respectively.

For comparison between the two models (statistical cell model and Suppan), Figure 12 shows the local mole fractions calculated through the statistical cell model (*p*_2_) and Suppan’s model (*y*_2_) as a function of the bulk mole fraction of ethanol and methanol, respectively. As can be observed, this figure emphasizes a very good agreement between the two models.

## 4. Materials and Methods

The spectrally grade alcohols were purchased from Merck Company and the binary solvent mixtures were realized step by step with molar fractions of 0.05. Bi-distilled water was prepared in our labs. The weighing was repeated twice in order to assure a good precision, having in mind the very small quantities used for each experiment. The weighing was made with a balance having a precision of 0.005 g.

**Figure 13:**
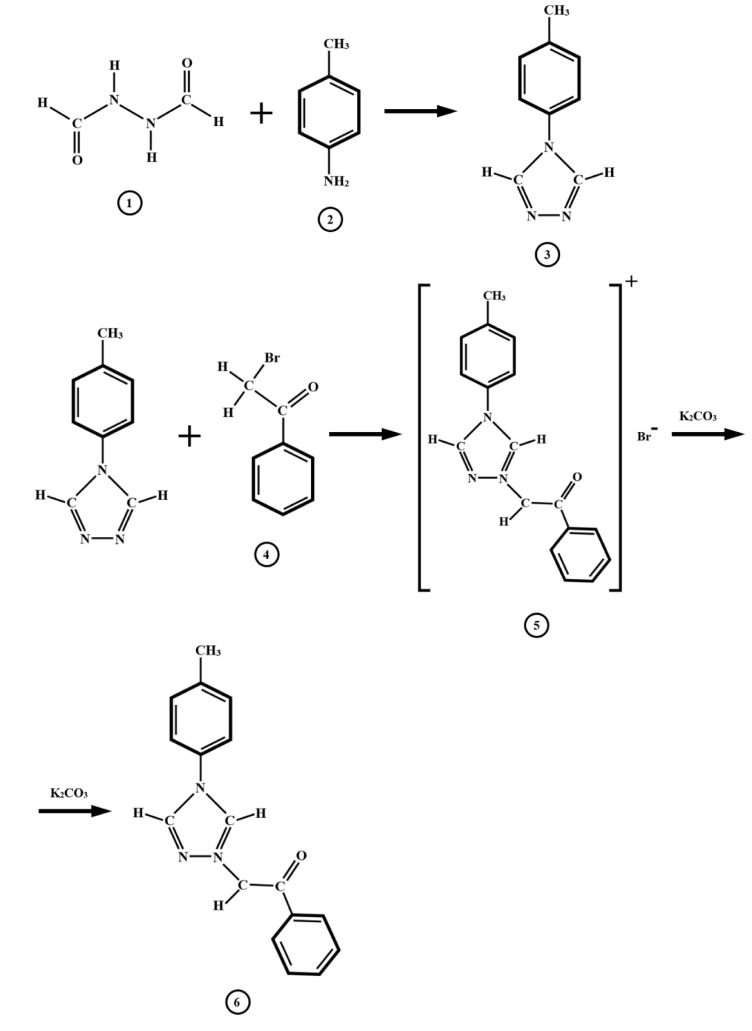
Schematic of the chemical reactions by which 4-tolyl-1,2,4-triazol-1-ium phenacylid (TTPY) was obtained (**1**—1,2-Diformylhydrazine, **2**—*p*-Toluidine, **3**—*p*-Tolyl-1,2,4-triazol-1-ium, **4**—2-Bromoacetophenone, **5**—TTPY salt, and **6**—TTPY).

The ylids TTPY and TTNPY were prepared [1,2] in the Organic Chemistry Labs of Alexandru Ioan Cuza University and verified from the purity point of view by quantitative elemental analysis, Fourier transform infrared (FT-IR) spectroscopy, and ^1^H nuclear magnetic resonance (NMR) spectroscopy. The salt method was used to prepare TTPY and TTNPY, the chemical reactions being schematically shown for TTPY in Figure 13; 0.05 mol of 1,2-diformylhydrazine mixed with 0.05 mol of *p*-Toluidine were refluxed for 8 h. Then, the mixture was kept in room temperature for 24 h. The obtained compound (*p*-Tolyl-1,2,4-triazol-1-ium) was dissolved in 50 mL of chloroform, while 0.05 mol of 2-Bromoacetophenone was dissolved in 50 mL of benzene. The two solutions were mixed, and potassium carbonate was added. The reaction was considered complete when the pH of the solution reached the value 8–8.5. The mixture was then kept at room temperature for 24 h. The white precipitate was filtered and then recrystallized from ethanol. TTNPY was obtained by the same procedure with one exception: 2-Bromo-4′-nitroacetophenone was used instead of 2-Bromoacetophenone. 1,2-diformylhydrazine, *p*-Toluidine, 2-Bromoacetophenone, and 2-Bromo-4′-nitroacetophenone were purchased from Sigma-Aldrich (now Merck).

To obtain the solutions of the two studied ylids, initially, two diluted solutions (10^−4^ mol/L concentration) were made: ylid + water and ylid + ethanol/ylid + methanol. Then, the two solutions were mixed in such a way that the molar fractions of the two solvents in the mixtures varied with a step of 0.05 (as it was made in the experiments for determining the KAT parameters, described in [20]).

The electronic absorption spectra were recorded with a Specord UV Vis Carl Zeiss Jena spectrophotometer with a data acquisition system, at a temperature *T* = 295.15 K. The wavenumber corresponding to the maximum of the electronic absorption band was determined by the first derivative method, after smoothing of the spectra.

## 5. Conclusions

As basic compounds, the studied ylids participate in specific interactions of the hydrogen bond types, and the formed complexes are subjected to the global action of the binary hydroxylic solvent mixture.

The specific interactions in binary solvent mixtures of TTNPY are enhanced by the presence of -NO_2_ substituent. The delocalization of the electronic charge on the oxygen atoms facilitates the proton addition on the carbanion, increasing the contribution of the specific interactions to the total spectral shift of TTNPY visible electronic absorption band.

The computed contribution of each type of interaction to the total shift of the ylid’s electronic absorption band is estimated in this paper based on the KAT parameters of the binary hydroxylic solvent mixtures, and show the universal interaction important contribution to the spectral shift.

The difference *w*_2_ − *w*_1_ between the energies corresponding to the hydrogen bonds in molecular pairs of the types TTPY-water, TTNPY-water, and ylid (TTPY/TTNPY)-alcohol (ethanol/methanol) is estimated here based on the statistical cell model of the binary solvent mixture solutions. The results reported in this paper are important, since cycloimmonium ylids, used in situ, are precursors in obtaining new heterocycle compounds in various domains.

For comparison, Suppan’s model was approached to investigate the preferential solvation of solutes in the binary solvent mixtures. The obtained results are in very good agreement with those obtained through statistical cell model.

## Figures and Tables

**Table 1:** Molar water composition (*x*_1_), Kamlet–Abboud–Taft parameters (π*, *β*, *α*) of binary solvent mixture water (1) + ethanol (2) [20], and wavenumbers ($\bar{\nu }$ (cm^−1^)) in the maximum of the visible electronic absorption band of 4-tolyl-1,2,4-triazol-1-ium phenacylid (TTPY) and 4-tolyl-1,2,4-triazol-1-ium-4′-nitro-phenacylid (TTNPY), respectively.

*x*_1_	π*	*α*	*β*	$\ln\frac{x_{1}}{1-x_{1}}$	$\bar{\nu }$		$\ln\frac{p_{1}}{1-p_{1}}$	
					TTPY	TTNPY	TTPY	TTNPY
0.000	0.51	0.98	0.83	-	26,670	27,690	-	-
0.050	0.54	0.97	0.83	−2.94	26,680	27,720	−3.56	−2.56
0.100	0.57	0.96	0.84	−2.20	26,690	27,748	−2.83	−1.83
0.150	0.60	0.94	0.83	−1.73	26,700	27,778	−2.40	−1.33
0.200	0.63	0.93	0.83	−1.39	26,718	27,810	−1.87	−0.92
0.250	0.65	0.93	0.83	−1.10	26,730	27,840	−1.61	−0.59
0.300	0.68	0.92	0.82	−0.85	26,748	27,868	−1.29	−0.31
0.350	0.70	0.91	0.81	−0.62	26,760	27,896	−1.10	−0.04
0.400	0.73	0.91	0.8	−0.41	26,780	27,920	−0.82	0.19
0.450	0.75	0.89	0.79	−0.20	26,800	27,940	−0.57	0.39
0.500	0.77	0.90	0.79	0.00	26,818	27,958	−0.36	0.57
0.550	0.80	0.89	0.78	0.20	26,830	27,980	−0.22	0.80
0.600	0.82	0.89	0.77	0.41	26,850	28,000	0.00	1.04
0.650	0.85	0.89	0.77	0.62	26,877	28,020	0.30	1.30
0.700	0.90	0.88	0.74	0.85	26,898	28,038	0.55	1.58
0.750	0.94	0.86	0.71	1.10	26,920	28,040	0.82	1.61
0.800	1.00	0.87	0.67	1.39	26,940	28,066	1.10	2.15
0.825	1.03	0.87	0.66	1.55	26,950	28,070	1.25	2.25
0.850	1.06	0.90	0.64	1.73	26,964	28,080	1.49	2.56
0.875	1.09	0.92	0.61	1.95	26,978	28,088	1.78	2.90
0.900	1.11	0.97	0.59	2.20	26,988	28,090	2.02	3.00
0.925	1.12	1.03	0.56	2.51	26,995	28,095	2.23	3.30
0.950	1.13	1.11	0.54	2.94	27,008	28,102	2.73	3.94
0.975	1.13	1.18	0.52	3.66	27,012	28,105	2.94	4.42
1.000	1.13	1.26	0.5	-	27,030	28,110	-	-

**Table 2:** Molar water composition (*x*_1_), Kamlet–Abboud–Taft parameters (π*, *β*, *α*) of binary solvent mixture water (1) + methanol (2) [20], and wavenumbers ($\bar{\nu }$ (cm^−1^)) in the maximum of the visible electronic absorption band of 4-tolyl-1,2,4-triazol-1-ium phenacylid (TTPY) and 4-tolyl-1,2,4-triazol-1-ium-4′-nitro-phenacylid (TTNPY), respectively.

*x*_1_	π*	*α*	*β*	$\ln\frac{x_{1}}{1-x_{1}}$	$\bar{\nu }$		$\ln\frac{p_{1}}{1-p_{1}}$	
					TTPY	TTNPY	TTPY	TTNPY
0.000	0.58	1.14	0.74	-	26,710	27,890	-	-
0.050	0.61	1.13	0.74	−2.94	26,738	27,908	−2.34	−2.42
0.100	0.64	1.12	0.74	−2.20	26,758	27,922	−1.73	−1.77
0.150	0.66	1.10	0.74	−1.73	26,780	27,938	−1.27	−1.28
0.200	0.70	1.09	0.74	−1.39	26,800	27,950	−0.94	−0.98
0.250	0.73	1.07	0.74	−1.10	26,820	27,962	−0.65	−0.72
0.300	0.76	1.06	0.74	−0.85	26,840	27,979	−0.38	−0.39
0.350	0.78	1.04	0.72	−0.62	26,860	27,986	−0.13	−0.26
0.400	0.82	1.04	0.72	−0.41	26,880	28,000	0.13	0.00
0.450	0.85	1.02	0.71	−0.20	26,898	28,016	0.35	0.29
0.500	0.88	1.03	0.7	0.00	26,910	28,024	0.51	0.44
0.550	0.91	1.02	0.68	0.20	26,928	28,038	0.76	0.72
0.600	0.95	1.01	0.66	0.41	26,940	28,050	0.94	0.98
0.650	0.98	1.01	0.65	0.62	26,952	28,060	1.13	1.22
0.700	1.01	1.01	0.63	0.85	26,968	28,070	1.43	1.50
0.750	1.04	1.02	0.61	1.10	26,982	28,080	1.73	1.85
0.800	1.06	1.06	0.59	1.39	26,996	28,086	2.13	2.10
0.825	1.08	1.07	0.58	1.55	27,000	28,088	2.27	2.20
0.850	1.09	1.09	0.56	1.73	27,008	28,092	2.61	2.42
0.875	1.10	1.12	0.55	1.95	27,012	28,098	2.82	2.85
0.900	1.11	1.13	0.54	2.20	27,016	28,100	3.08	3.04
0.925	1.11	1.17	0.52	2.51	27,022	28,104	3.66	3.57
0.950	1.12	1.19	0.52	2.94	27,024	28,106	3.96	3.99
0.975	1.12	1.22	0.51	3.66	27,026	28,107	4.37	4.28
1.000	1.14	1.23	0.49	-	27,030	28,110	-	-

**Table 3:** Regression parameters in relation $\bar{\nu }\left({\mathrm{cm}}^{-1}\right)={\bar{\nu }}_{0}\left({\mathrm{cm}}^{-1}\right)+C_{1}\pi^{*}+C_{2}\alpha +C_{3}\beta$ and the regression coefficient (R) for the binary solvent mixtures solutions of 4-tolyl-1,2,4-triazol-1-ium phenacylid (TTPY) and 4-tolyl-1,2,4-triazol-1-ium-4′-nitro-phenacylid (TTNPY), respectively, with parameters from Table 1 and Table 2.

Binary Solvent Mixture	Ylid	${\bar{\nu }}_{0}\pm \Delta {\bar{\nu }}_{0}$	$C_{1}\pm \Delta C_{1}$	$C_{2}\pm \Delta C_{2}$	$C_{3}\pm \Delta C_{3}$	R
Water + Ethanol	TTPY	25,949 ± 231	696 ± 72	108 ± 63	291 ± 160	0.99178
Water + Ethanol	TTNPY	24,915 ± 310	1485 ± 96	450 ± 84	1880 ± 214	0.98811
Water + Methanol	TTPY	25,583 ± 171	804 ± 47	187 ± 54	612 ± 112	0.99179
Water + Methanol	TTNPY	27,279 ± 99	511 ± 27	79 ± 31	309 ± 65	0.99806

**Table 4:** Contribution of each type of interaction in solutions of 4-tolyl-1,2,4-triazol-1-ium phenacylid (TTPY) in the binary hydroxylic solvents mixtures.

*x*_1_	Water + Ethanol			Water + Methanol		
	*C*_1_π* (%)	*C*_2_*α* (%)	*C*_3_*β* (%)	*C*_1_π* (%)	*C*_2_*α* (%)	*C*_3_*β* (%)
0.000	50.54	15.07	34.39	41.18	18.85	39.96
0.050	52.04	14.51	33.45	42.48	18.33	39.19
0.100	53.26	13.92	32.82	43.72	17.82	38.45
0.150	54.90	13.35	31.76	44.62	17.32	38.06
0.200	56.18	12.87	30.95	46.15	16.74	37.11
0.250	56.95	12.64	30.41	47.34	16.16	36.50
0.300	58.34	12.25	29.41	48.42	15.73	35.86
0.350	59.33	11.97	28.71	49.69	15.43	34.88
0.400	60.54	11.71	27.75	50.94	15.05	34.02
0.450	61.55	11.33	27.11	52.22	14.60	33.18
0.500	62.10	11.26	26.64	53.26	14.52	32.22
0.550	63.28	10.92	25.80	54.66	14.27	31.07
0.600	64.06	10.79	25.15	56.30	13.94	29.75
0.650	64.88	10.54	24.58	57.32	13.76	28.92
0.700	66.86	10.15	22.99	58.57	13.64	27.79
0.750	68.60	9.74	21.67	59.72	13.64	26.64
0.800	70.66	9.54	19.80	60.38	14.06	25.56
0.825	71.48	9.37	19.15	61.00	14.08	24.92
0.850	72.24	9.52	18.24	61.59	14.34	24.07
0.875	73.26	9.60	17.14	61.83	14.66	23.51
0.900	73.64	9.99	16.37	62.22	14.75	23.02
0.925	73.98	10.56	15.47	62.43	15.33	22.24
0.950	73.95	11.27	14.78	62.48	15.46	22.06
0.975	73.83	11.96	14.21	62.50	15.86	21.65
1.000	73.63	12.74	13.62	63.36	15.92	20.72

**Table 5:** Contribution of each type of interaction in solutions of 4-tolyl-1,2,4-triazol-1-ium-4′-nitro-phenacylid (TTNPY) in binary hydroxylic solvents mixtures.

*x*_1_	Water + Ethanol			Water + Methanol		
	*C*_1_π* (%)	*C*_2_*α* (%)	*C*_3_*β* (%)	*C*_1_π* (%)	*C*_2_*α* (%)	*C*_3_*β* (%)
0.000	27.45	15.98	56.57	48.22	14.58	37.21
0.050	28.65	15.60	55.76	49.54	14.11	36.35
0.100	29.62	15.12	55.27	50.80	13.67	35.53
0.150	30.99	14.72	54.29	51.69	13.25	35.05
0.200	32.10	14.36	53.54	53.22	12.75	34.03
0.250	32.78	14.21	53.00	54.39	12.26	33.35
0.300	34.05	13.96	51.99	55.45	11.90	32.65
0.350	34.97	13.78	51.25	56.71	11.63	31.66
0.400	36.16	13.66	50.18	57.93	11.30	30.77
0.450	37.13	13.35	49.52	59.18	10.92	29.90
0.500	37.69	13.35	48.96	60.20	10.84	28.96
0.550	38.88	13.11	48.00	61.56	10.61	27.82
0.600	39.71	13.06	47.22	63.14	10.33	26.53
0.650	40.58	12.88	46.55	64.12	10.16	25.72
0.700	42.78	12.68	44.54	65.31	10.05	24.64
0.750	44.77	12.41	42.82	66.42	10.02	23.56
0.800	47.35	12.48	40.17	67.09	10.32	22.59
0.825	48.37	12.38	39.25	67.70	10.32	21.99
0.850	49.46	12.73	37.81	68.28	10.50	21.22
0.875	50.91	13.02	36.07	68.54	10.73	20.73
0.900	51.60	13.67	34.73	68.93	10.79	20.28
0.925	52.31	14.58	33.12	69.18	11.22	19.60
0.950	52.55	15.64	31.80	69.24	11.32	19.44
0.975	52.65	16.66	30.68	69.30	11.61	19.09
1.000	52.68	17.80	29.52	70.13	11.64	18.23

**Table 6:** Characteristics of the lines $\ln\frac{p_{1}}{1-p_{1}}=m\ln\frac{x_{1}}{1-x_{1}}+n$ for the studied binary solvent mixtures solutions.

Solution	m	Δm	n	Δ*n*	R	*w*_2_ − *w*_1_ (kJ/mol)
TTPY + Water + Ethanol	1.0525	0.0164	−0.4166	0.0282	0.9947	−1.033 ± 0.070
TTNPY + Water + Ethanol	1.0881	0.0101	0.6034	0.0173	0.9981	1.496 ± 0.043
TTPY + Water + Methanol	1.0888	0.0162	0.6046	0.0278	0.9952	1.499 ± 0.069
TTNPY + Water + Methanol	1.0922	0.0166	0.5693	0.0285	0.9949	1.411 ± 0.071

**Table 7:** Results obtained through Suppan’s model.

Solution	Intercept	Slope	Index of Preferential Solvation *Z*	*R*
TTPY + Water + Ethanol	0.7491 ± 0.0699	1.8064 ± 0.0151	−0.880	0.9984
TTNPY + Water + Ethanol	0.7538 ± 0.0176	0.5721 ± 0.0038	0.276	0.9990
TTPY + Water + Methanol	1.1490 ± 0.0438	0.6198 ± 0.0094	0.617	0.9947
TTNPY + Water + Methanol	1.6482 ± 0.0511	0.9609 ± 0.0110	0.540	0.9970

## Data Availability

The data presented in this study are available on request from the corresponding author.

## Sample Availability

Not available.

## References

[1] Petrovanu M., Luchian C., Surpateanu G., Barboiu V. (1979). 1,2,4-Triazolium ylures I. Synthèsis et stréréochimie des réactions de cycloaddition aux composés á liaison ethylenique active. Rev. Roum. Chim..

[2] Surpateanu G., Caea N., Sufletel L., Grandclaudon P. (1995). Synthesis characterization of new azatriazolium ylids. Rev. Roum. Chim..

[3] Lars-Flörl C. (2011). Triazole antifungal agents in invasive fungal infections. A comparative review. Drugs.

[4] Satish Kumar S., Kavitha H.P. (2013). Synthesis and biological applications of triazole derivatives—A review. Mini Rev. Org. Chem..

[5] Al-Omar M.A., Al-Abdullah E.S., Shehata I.A., Habib E.E., Ibrahim T.M., El-Emam A.A. (2010). Synthesis, antimicrobial and anti-inflammatory activities of novel 5-(1-Adamantyl)-4-arylideneamino-3-mercapto-1,2,4-triazoles and related derivatives. Molecules.

[6] Chen X., Shi Y.-M., Huang C., Xia S., Yang L.-J., Yang X.-D. (2016). Novel dibenzo[b,d]furan–1H-1,2,4-triazole derivatives: Synthesis and antitumor activity. Anti-Cancer Agents Med. Chem..

[7] Shah M.H., Mhasalkar M.Y., Patki V.M., Deliwala C.V., Sheth U.K. (1969). New 1,2,4(H)-triazole derivatives as diuretic agents. J. Pharm. Sci..

[8] Alrawashdeh M.S.M. (2018). Determination of antimicrobial activity of some 1,2,4-triazole derivatives. Regul. Mech. Biosyst..

[9] Karczmarzyk Z., Swatko-Ossor M., Wysocki W., Drozd M., Ginalska G., Pachuta-Stec A., Pitucha M. (2020). New applications of 1,2,4-triazole derivatives as antitubercular agents. Structure, in vitro screening and docking studies. Molecules.

[10] Dorohoi D.O., Partenie H. (1993). The spectroscopy of cycloimmonium ylides. J. Mol. Struct..

[11] Dorohoi D.O. (2004). Electronic spectroscopy of N-Ylid. J. Mol. Struct..

[12] Dorohoi D.O., Dimitriu D.G., Dimitriu M., Closca V. (2013). Specific interactions in N-ylid solutions, studied by nuclear magnetic resonance and electronic absorption spectroscopy. J. Mol. Struct..

[13] Melniciuc-Puica N., Barboiu V., Filoti S., Dorohoi D.O. (2004). Reactivity of some 1 (*N*)-[(*para*-R_2_)-phenacyl]-4(*N*)-[(*para*-R_1_)-phenyl]-1,2,4-triazolium methylides by UV-VIS, IR and NMR spectra and molecular modeling. Spectrosc. Lett..

[14] Puica-Melniciuc N., Ivan L.M., Closca V., Dorohoi D.O. (2019). Electro-optical and spectral comparative study of some triazolium methylids with biomedical applications. Rev. Chim..

[15] Closca V., Melniciuc-Puica N., Dorohoi D.O. (2014). Specific interactions in hydroxyl ternary solutions of three carbanion mono-substituted 4’-tolyl-1,2,4-triazol-1-ium-4-R- phenacylids studied by visible electron spectra. J. Mol. Liq..

[16] Closca V., Puica Melniciuc N., Dorohoi D.O., Benchea A.C. (2014). Intermolecular interactions in ternary solutions of some 1,2,4 triazolium ylids studied by spectral means. Proc. SPIE.

[17] Closca V., Puica Melniciuc N., Closca M., Avadanei I.M., Dorohoi D.O. (2018). Spectral study of (4’-phenyl)-1,2,4-triazol-1-ium-phenacylid (PTPhY) in ternary solutions. Ukr. J. Phys..

[18] Kamlet M.J., Abboud J.L.M., Abraham M.H., Taft R.W. (1983). Linear solvation energy relationships. 23. A comprehensive collection of the solvatochromic parameters, π*, α, and β, and some methods for simplifying the generalized solvatochromic equation. J. Org. Chem..

[19] Reichardt C. (2003). Solvents and Solvent Effects in Organic Chemistry.

[20] Buhvestov U., Rived F., Ràfols C., Bosch E., Rosés M. (1998). Solute-solvent and solvent-solvent interactions in binary solvent mixtures. Part 7. Comparison of the enhancement of the water structure in alcohol-water mixtures measured by solvatochromic indicators. J. Phys. Org. Chem..

[21] Ivan L.M., Dimitriu D.G., Gritco-Todirascu A., Morosanu A.C., Dorohoi D.O., Cheptea C. (2020). Excited state dipole moment of two pyridazinium–p-nitro-phenacylids estimated from solvatochromic study. Spectrosc. Lett..

[22] Dorohoi D.O., Creanga D.E., Dimitriu D.G., Morosanu A.C., Gritco-Todirascu A., Mariciuc G.G., Puica Melniciuc N., Ardelean E., Cheptea C. (2020). Computational and spectral means for characterizing the intermolecular interactions in solutions and for estimating excited state dipole moment of solute. Symmetry.

[23] Dulcescu Oprea M.M., Melniciuc Puica N., Gritco-Todirascu A., Dorohoi D.O. (2020). Spectral study of two carbanion monosubstituted 4’-phenyl-1,2,4-triazol-1-ium phenacylids in binary protic solvents. Rev. Chim..

[24] Pop V., Dorohoi D.O., Delibas M. (1986). Consideration on the statistic model of the intermolecular interactions in ternary solutions. An. Stiint. Univ. Al. I. Cuza, Iasi, s.Ib, Fizica.

[25] Dorohoi D.O., Pop V. (1987). Spectral shifts in the electronic absorption spectra of some cycloimmonium ylids in ternary solutions. An. Stiint. Univ. Al. I. Cuza, Iasi, s.Ib, Fizica.

[26] Dorohoi D.O., Avadanei I.M., Postolache M. (2008). Characterization of the solvation spheres of some dipolar spectrally active molecules in binary solvents. Optoelectron. Adv. Mater..

[27] Babusca D., Benchea A.C., Dimitriu D.G., Dorohoi D.O. (2016). Solvatochromic characterization of Sudan derivatives in binary and ternary solutions. Anal. Lett..

[28] Avadanei M., Dorohoi D.O. (2012). Interaction energy in pairs of pyridazinium ylids-solvent molecules estimated by spectral means within the cell ternary solution model. Ukr. J. Phys..

[29] Sasirekha V., Vanelle P., Terme T., Ramakrishnan V. (2008). Solvatochromism and preferential solvation of 1,4-dihydroxy-2,3-dimethyl-9,10 anthraquinone by UV-vis absorption and laser induced fluorescence measurements. Spectrochim. Acta A.

[30] Suppan P. (1987). Local polarity of solvent mixtures in the field of electronically excited molecules and exciplexes. J. Chem. Soc. Faraday Trans..

[31] Lerf C., Suppan P. (1992). Hydrogen bonding and dielectric effects in solvatochromic shifts. J. Chem. Soc. Faraday Trans..

[32] Henseler A., von Raumer M., Suppan P. (1996). Observation of dielectric enrichment upon the formation of benzophenone radical anion in a binary solvent mixture. J. Chem. Soc. Faraday Trans..

[33] Papadakis R. (2016). Preferential solvation of a highly medium responsive Pentacyanoferrate(II) complex in binary solvent mixtures: Understanding the role of dielectric enrichment and the specificity of solute-solvent interactions. J. Phys. Chem. B.

[34] Papadakis R., Deligkiozi I., Nowak K.E. (2019). Study of the preferential solvation effects in binary solvent mixtures with the use of intensely solvatochromic azobenzene involving [2] rotaxane solutes. J. Mol. Liq..

[35] Malik P.K., Tripathy M., Kajjam A.B., Patel S. (2020). Preferential solvation of *p*-nitroaniline in a binary mixture of chloroform and hydrogen bond acceptor solvents: The role of specific solute-solvent hydrogen bonding. Phys. Chem. Chem. Phys..

[36] Malik P.K., Tripathy M., Patel S. (2020). D-π-A molecular probe to unveil the role of solute-solvent hydrogen bonding in solvatochromism, location specific preferential solvation and synergistic effect in binary mixtures. ChemistrySelect.

[37] Van S.-P., Hammond G.S. (1978). Amine quenching of aromatic fluorescence and fluorescent exciplexes. J. Am. Chem. Soc..

[38] Dulcescu-Oprea M.M., Morosanu A.C., Dimitriu D.G., Gritco-Todirascu A., Dorohoi D.O., Cheptea C. (2021). Solvatochromic study of pyridinium acetyl benzoyl methylid (PABM) in ternary protic solutions. J. Mol. Struct..

